# Abusive consumption of alcoholic beverages: results from COVITEL, the Telephone Survey of Risk and Protective Factors for Noncommunicable Chronic Diseases, 2022 and 2023

**DOI:** 10.1590/1980-549720250009

**Published:** 2025-02-24

**Authors:** Roberta de Oliveira Santos, Fernando César Wehrmeister, Pedro Hallal, Eduardo Ribes Kohn, Luciana Monteiro Vasconcelos Sardinha

**Affiliations:** I Vital Strategies, Department of Noncommunicable Chronic Diseases – São Paulo (SP), Brazil.; II Universidade Federal de Pelotas, Centro de Equidade – Pelotas (RS), Brazil.; III University of Illinois, Department of Kinesiology and Community Health – Urbana-Champaign, United States of Amrecia.; IV Universidade Federal de Pelotas, Graduate Program in Physical Education – Pelotas (RS), Brazil.

**Keywords:** Alcohol drinking, Brazil, Health surveys, Cross-sectional study

## Abstract

**Objective:**

To estimate the prevalence of abusive alcohol consumption, drinking and driving habits and reports of alcohol consumption comparing the first quarters of 2022 and 2023.

**Methods:**

A cross-sectional study, with data from the Telephone Survey of Risk Factors for Chronic Noncommunicable Diseases, 2022 and 2023. The study sample included 9 thousand people each year collected using random digit dialing and dialing methods direct distance (DDD) on mobile and fixed telephone lines. Self-reported variables for alcohol abuse, drinking and driving habits, and alcohol consumption were analyzed.

**Results:**

There was no significant change in the prevalence of alcohol abuse in the first quarters of 2022 and 2023. However, differences were observed in drinking and driving behavior, with a reduction in prevalence among those aged 18 to 24 years (9.6% (95%CI 4.4–19.8) to 2.2% (95%CI 1.4–3.6) and increased behavior among those with 12 or more years of education (from 6.9% (95%CI 5.5–8.7) to 11.9% (95%CI 10,3–13,6). Male individuals had a higher prevalence of alcohol consumption, alcohol abuse and drinking and driving habits in all analyzed breakdowns.

**Conclusion:**

The Brazilian policy to reduce the consumption of alcoholic beverages and the Sustainable Development Goals must be treated as a priority in Brazil.

## INTRODUCION

Alcohol consumption refers to the ingestion of beverages containing ethanol and is recognized as one of the primary risk factors for noncommunicable diseases, violence, and accidents, directly or indirectly contributing to over three million deaths globally each year.^1^ Beyond its impact on morbidity and mortality, alcohol consumption has significant economic consequences for health systems and social services, including costs related to early retirement, workplace absenteeism, and reduced productivity.^1–4^ Estimates indicate that global per capita alcohol consumption among individuals aged 15 years old and older increased from 5.5 liters of pure alcohol in 2005 to 6.4 liters in 2016.^1^ In 2018, approximately 2.3 billion people were reported to have consumed alcohol^1^.

Data from the 2013 and 2019 National Health Survey indicate an increase in alcohol consumption among the Brazilian population^5^. The same survey reveals that men consume more alcohol than women; however, the increase in consumption in the period from 2013 to 2019 was more pronounced among women^5^. Additionally, a rise in alcohol abuse was observed between 2013 and 2019^6^. Findings from the CONVID Behaviors study in Brazil highlighted an increase in alcohol abuse during the first wave of COVID-19 in 2020^7–9^. Furthermore, data from the Fiocruz report on alcoholic beverages in Brazil show the consumption of 13 million liters of beer and 2.6 million liters of distilled beverages in 2021^10^.

Previous studies on alcohol consumption in Brazil have provided significant insights for monitoring this behavioral pattern. In this context, the present study aimed to expand the knowledge base on alcohol consumption among the Brazilian population by comparing binge drinking and drinking and driving behaviors during the first quarters of 2022 and 2023, considering socioeconomic and demographic characteristics.

## METHODS

This is a cross-sectional, population-based study utilizing secondary data from the Telephone Survey of Risk Factors for Non-Communicable Chronic Diseases (*Inquérito Telefônico de Fatores de Risco para Doenças Crônicas não Transmissíveis* – Covitel) conducted in 2022 and 2023. Covitel is a national survey carried out by Vital Strategies Brasil and Universidade Federal de Pelotas (UFPel), with support from Umane, the Ibirapitanga Institute, and the Brazilian Association of Public Health (*Associação Brasileira de Saúde Coletiva* – Abrasco). The Covitel project received approval from the Research Ethics Committee of the School of Physical Education at Universidade Federal de Pelotas (Opinion No. 5.125.635, issued on November 25, 2021).

The Covitel sample was segmented into different strata based on geographic region (Northeast, North, Southeast, South, and Central-West), gender (male and female), age range (18–34; 35–49, and 50 years old or older), and education level (0 to 11 years and 12 years or more). To calculate the sample weights and ensure representation of the Brazilian population and its regions, data from the IBGE Automatic Recovery System (Sidra; Table 3450, based on the 2010 Demographic Census sample) were utilized. As a result, the population was estimated across 60 strata, considering the combination of geographic region (5), gender (2), age range (3), and education level.

Sampling was conducted in two stages. The first stage involved creating a telephone registry of residential and mobile phone lines using the random digit dialing method, proportional to the number of lines per Direct Distance Dialing (DDD) code in each region of the country. The second stage consisted of the random selection of individuals. For each eligible landline, individuals were selected from a list of all household residents aged 18 years old or older, sorted in ascending order by age. For mobile phone lines, the person responsible for the line was interviewed if they were 18 years old or older. Informed consent was obtained orally at the time of the interview.

The sample consisted of 1,800 individuals per macro-region, totaling 9,000 individuals for each year studied, with half allocated to landlines and the other half to cell phones. This sample size allows for the estimation of the frequency of any risk factor in the studied population with a 95% confidence interval and a maximum margin of error of approximately three percentage points. For gender-specific estimates, a maximum margin of error of around four percentage points is expected, assuming similar proportions of men and women in the final sample. Since Covitel selects participants based on the area code associated with the telephone line, which is equivalent to cluster sampling, this factor was accounted for in the analysis along with the sample weights. Consequently, the derived estimates were adjusted for both the cluster effect and the described weighting to produce values representative of the regional and national populations. Details on the study methodology have been previously described by Hallal et al.^11^.

The analyses of the prevalence of alcohol abuse and drinking and driving between the first quarters of 2022 and 2023 were conducted based on the following variables:

- Alcohol abuse — those who reported having consumed 4 doses (women) or 5 doses (men) on a single occasion, in the 30 days prior to the interview;
- Drinking and driving — those who reported that, regardless of the amount, they usually drove after consuming alcoholic beverages.

In addition to the main outcomes, the prevalence of reported alcohol consumption at least once in the past month was analyzed using the retrospective variable, "*Before the onset of the pandemic, did you usually consume alcoholic beverages?*" and the variable, "*Currently, do you usually consume alcoholic beverages?*" from the 2022 and 2023 Covitel surveys.

For the sociodemographic and lifestyle analysis, the following characteristics were considered:

- Gender (male and female);
- Age range (18-24; 25-34; 35-44; 45-54; 55-64 and 65years old or older);
- Regions of Brazil (North, Northeast, Central-West, Southeast, South);
- Education level (0-8; 9-11 and 12 or more);
- Skin color (white; black and brown and others).

Relative frequency measures (prevalence) and their respective 95% confidence intervals were calculated. Sampling weights were imputed using the survey module for complex data analysis, based on data from the 2010 Brazilian Census conducted by the Brazilian Institute of Geography and Statistics (*Instituto Brasileiro de Geografia e Estatística* – IBGE). The sample was stratified by geographic region (Northeast, North, Southeast, South, and Central-West), gender (male and female), age (18–34; 35–49, and 50 years old or older), and education (0–11 years and 12 or more years of education). Adjustments were made for age (the 18–19 age group corresponds to 2/5 of the IBGE category of 15–19 years) and education (the 0–11 years of education category was created for those with less than a high school education in the IBGE categories). Significant changes in the prevalence of the indicators were determined by non-overlapping confidence intervals. Data analysis was performed using Stata statistical software, version 16 (Stata Corp., College Station, TX, USA), and the figures were generated using the Equiplot Creator Tool (International Center for Equity in Health — UFPel).

## RESULTS

The total number of individuals interviewed in each phase of the Covitel study (2022 and 2023) was 9,000, with equal distribution across the five regions of the country. No significant difference in alcohol abuse was observed between the first quarter of 2022 and 2023. The overall prevalence of reported drinking and driving was higher in the first quarter of 2023 (2.6%, 95%CI 1.9–3.3) compared to the first quarter of 2022 (4.9%, 95%CI 3.7–6.2). Subgroups with an education level of ≥12 years showed a higher prevalence in 2023 (11.9%, 95%CI 10.3–13.6) compared to 2022 (6.9%, 95%CI 5.5–8.7). The age group 18–24 years experienced a reduction in prevalence, from 9.6% (95%CI 4.4–19.8) in the first quarter of 2022 to 2.2% (95%CI 1.4–3.6) in the first quarter of 2023 (Table 1).

**Table 1:** Prevalence of excessive alcohol consumption and drinking and driving, comparing the first quarter of 2022 and 2023, according to demographic variables. Covitel, 2022 and 2023.

Sociodemographic and Lifestyle Characteristics		Excessive Alcohol Consumption				Drinking and Driving			
		2022		2023		2022		2023	
		%	9%CI	%	95%CI	%	95%CI	%	95%CI
**Gender**									
	Male	26.6	24.4–28.9	28.9	25.8–32.3	7.6	5.8–10.0	10.6	8.2–13.6
	Female	15.0	12.7–17.7	15.7	13.7–18.9	3.4	2.3–4.9	2.1	1.5–3.0
**Age range**									
	18 to 24 years	25.8	21.2–30.9	32.6	26.4–39.5	9.6	4.4–19.8	2.2 ↓	1.4–3.6
	25 to 34 years	26.9	23.2–31.0	27.0	22.5–32.0	6.0	3.8–9.3	8.7	6.0–12.3
	35 to 44 years	23.2	20.5–26.1	23.7	20.1–27.7	7.7	5.9–9.8	9.7	7.0–13.3
	45 to 54 years	20.1	17.2–23.4	23.6	19.5–28.3	5.1	3.5–7.4	6.7	4.5–10.0
	55 to 64 years	12.9	10.0–16.5	14.4	11.9–17.3	4.5	2.2–8.8	3.7	2.3–5.8
	65 years or more	5.5	4.3–7.1	5.1	4.0–6.4	2.2	0.8–5.4	1.5	0.9–2.4
**Region**									
	North	22.8	21.5–24.3	19.0	15.3–23.2	6.7	5.0–8.8	6.9	4.6–10.2
	Northeast	20.0	16.8–23.7	21.2	17.7–25.1	7.5	4.4–12.6	5.4	3.0–9.6
	Central-West	18.9	15.1–23.2	21.9	17.4–27.2	6.0	3.8–9.3	4.1	2.2–7.4
	Southeast	21.0	18.1–24.1	23.7	20.8–26.8	5.8	4.0–8.3	6.8	4.9–9.3
	South	20.3	17.0–24.1	20.6	18.3–23.1	5.1	2.9–8.8	6.8	4.5–10.3
**Education**									
	0 to 8 years	17.1	14.8–19.6	19.8	16.2–24.0	4.6	2.6–8.0	4.9	3.1–7.7
	9 to 11 years	22.5	19.8–25.5	22.9	19.5–26.7	7.6	5.0–11.4	4.6	2.9–7.2
	12 or more	26.6	24.3–29.1	26.6	25.0–28.2	6.9	5.5–8.7	11.9 ↑	10.3–13.6
**Skin color**									
	White	18.8	16.5–21.4	19.8	16.6–23.3	7.0	5.5–8.8	7.8	5.9–10.4
	Black and Brown	22.5	20.2–24.9	18.8	16.0–21.9	5.7	4.0–8.0	4.1	2.8–5.8
	Others	16.1	10.6–23.7	16.9	11.4–24.2	4.7	1.3–15.8	4.9	2.3–10.1
**Total**		20.6	18.9–22.4	22.1	20.2–24.0	2.6	1.9–3.3	4.9 ↑	3.7–6.2

↓ decrease in prevalence during the first quarter of 2023;

↑ increase in prevalence during the first quarter of 2023.

Indicators without a symbol indicate no change in the prevalence of drinking and driving or excessive alcohol consumption based on the overlap of confidence intervals.

Among the stratified groups, the prevalence of binge drinking was significantly higher among men in both 2022 (26.6%, 95%CI 24.4–28.9) and 2023 (28.9%, 95%CI 25.8–32.3), compared to women in 2022 (15%, 95%CI 12.7–17.7) and 2023 (15.7%, 95%CI 13.7–18.9). Individuals aged 65 years old or older had significantly lower prevalences of binge drinking compared to those in the <65 years old subgroups in both the first quarter of 2022 and 2023. Additionally, individuals with 0 to 8 years of schooling had significantly lower prevalences only in the first quarter of 2022 compared to those with a higher level of education (Table 1).

For the report of drinking and driving, the prevalence was significantly higher among men in 2022 (7.6%, 95%CI 5.8–10.0) and 2023 (10.6%, 95%CI 8.2–13.6), compared to women in 2022 (3.4%, 95%CI 2.3–4.9) and 2023 (2.1%, 95%CI 1.5–3.0). The most extreme age subgroups had lower prevalences of drinking and driving compared to the other subgroups, but only in 2023. Individuals with ≥12 years of education in 2023 had a significantly higher prevalence of drinking and driving (11.9%, 95%CI 10.3–13.6) compared to those with 9 to 11 years of education (4.6%, 95%CI 2.9–5.8) and those with 0 to 8 years of education (4.9%, 95%CI 2.9–7.2), as shown in Table 1.

**Figure 1:**
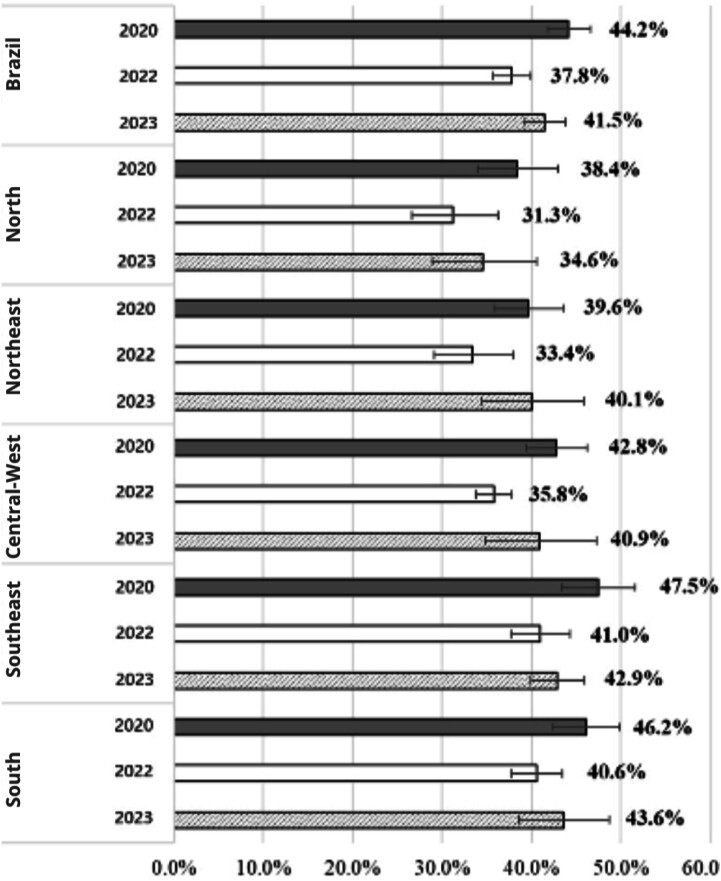
Prevalence of alcohol consumption by Brazil and regions of the country in the first quarter of 2020, the first quarter of 2022, and the first quarter of 2023. Covitel, 2022 and 2023.

**Figure 2:**
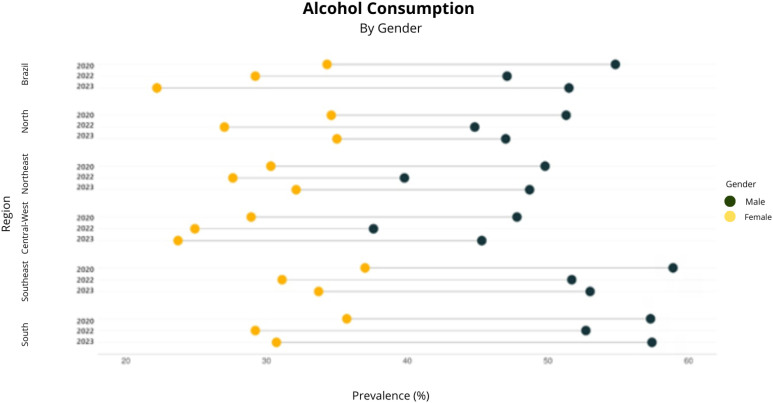
Prevalence of alcohol consumption by gender in the first quarter of 2020, the first quarter of 2022, and the first quarter of 2023. Covitel, 2022 and 2023.

**Figure 3:**
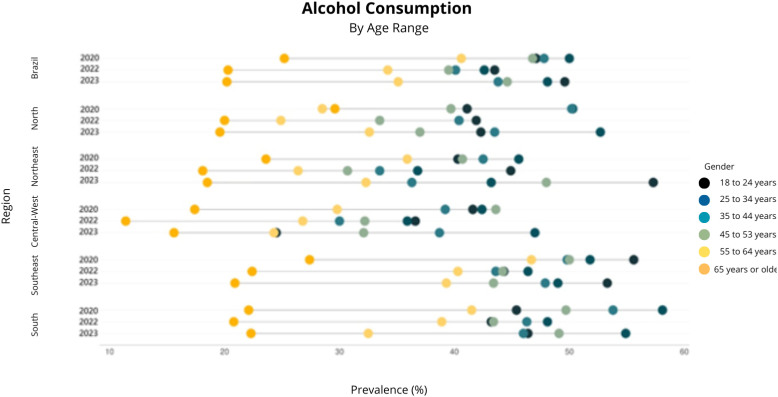
Prevalence of alcohol consumption by age range in the first quarter of 2020, the first quarter of 2022, and the first quarter of 2023. Covitel, 2022 and 2023.

**Figure 4:**
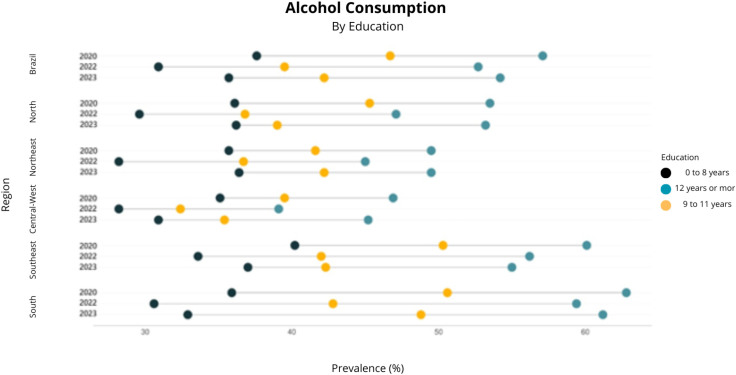
Prevalence of alcohol consumption by educational attainment in the first quarter of 2020, the first quarter of 2022, and the first quarter of 2023. Covitel, 2022 and 2023.

Reported alcohol consumption in Brazil, analyzed retrospectively in January 2020, showed a higher prevalence (44.2%, 95%CI 41.8–46.6) compared to 2022 (37.8%, 95%CI 35.7–40.0). However, the prevalence of this indicator in 2023 did not show a significant difference compared to the other two time points (41.5%, 95%CI 39.2–43.9) (Figures 1 to 4).

## DISCUSSION

This study analyzed the prevalence of binge drinking by comparing the first quarters of 2022 and 2023 in Brazil. No significant change was observed in the prevalence of binge drinking during the Covitel study periods. The literature suggests that individuals with this consumption profile are often less motivated to alter their behaviors due to the body's adaptation to the negative consequences associated with repeated intoxication^12,13^. In the United States, a cohort of more than 2 million individuals observed that binge drinking decreased from 15.5 to 14.6% during the first year of the pandemic, increased to 15.2% in the second year, and then decreased to 14.9% in the period from March 2022 to February 2023^14^, maintaining a degree of stability.

However, the similar prevalence observed in the alcohol abuse data from Covitel should not be considered a positive outcome, even though the period partially encompasses the COVID-19 pandemic. The observed period is relatively short, and the consequences of the pandemic may lead to an increase in this indicator. Evidence suggests that, in the years following traumatic events and economic recessions, alcohol consumption tends to rise as a result of stress and anxiety episodes^15–18^.

Alcohol consumption is considered a global health priority and is addressed within the Sustainable Development Goals (SDGs) under the objective of "strengthening the prevention and treatment of substance abuse, including drug abuse and the harmful use of alcohol"^19^. Prior to the pandemic, the World Health Organization (WHO) developed a technical package with five impact strategies aimed at helping governments reduce the harmful use of alcohol and its social, economic, and health consequences^20^. Brazil must address alcohol consumption with the importance it deserves for public health, in line with global recommendations. In this regard, the National Drug Policy Plan (2022-2027) can play a critical role in tackling the abusive use of alcohol in Brazil^21^.

Regarding the prevalence of drinking and driving in the first quarter of 2023, individuals aged 18 to 24 years showed a reduction in this indicator, while those with 12 or more years of education experienced an increase, compared to the period in 2022. There is no clear evidence to explain these changes within these subgroups. Vingilis and colleagues suggested that the COVID-19 pandemic had the potential to affect certain social groups differently^22^. One hypothesis for the decrease in drinking and driving among younger individuals is the loss of jobs and income, which imposed worse economic conditions on this subgroup. In contrast, individuals with 12 or more years of education may have been less economically affected by the pandemic. Data from the National Health Survey (*Pesquisa Nacional de Saúde* – PNS) show an association between drinking and driving and males with higher incomes^23^.

Previous studies have identified that one of the predictors of drinking and driving is the absence of punitive consequences^24,25^, meaning that individuals who engage in alcohol-impaired driving without being caught and penalized are more likely to continue this behavior^26^. Drivers with no prior history of drinking and driving who begin engaging in this behavior are also less likely to face punishment, which may further encourage the continuation of this risky practice^27^. Reducing global deaths and injuries from road accidents by half is a component of the Sustainable Development Goals^19^, which can be achieved, in part, through awareness-raising initiatives targeting drinking and driving.

The prevalence of reported alcohol consumption in Brazil was lower in the first quarter of 2022 compared to the pre-pandemic period in the first quarter of 2020. In contrast, the ConVid study in Brazil found that alcohol consumption increased during the pandemic compared to the pre-pandemic period^8^. Among adolescents, however, the ConVid study observed a significant decrease in the prevalence of alcohol consumption from the pre-pandemic period to the pandemic^28^. In the United States, adults consumed alcoholic beverages on average one additional day per month during the first year of the pandemic^29^. In Mexico and Colombia, the prevalence of alcohol consumption declined during the pandemic, with monthly reductions in the Alcohol Use Disorders Identification Test (AUDIT) score of 1.9% in Mexico and 1.5% in Colombia^30^.

During health crises, two opposing scenarios regarding alcohol consumption are possible. In the first, increased stress resulting from social isolation may lead to higher alcohol consumption. In the second, distancing measures can create barriers to accessing points of sale, loss of employment and income may reduce financial capacity for purchasing alcohol, and restrictions on social gatherings can limit opportunities for consumption^31,32^. The Covitel studies were conducted during the COVID-19 pandemic, making it possible to observe changes in consumption patterns during this period.

Men exhibited higher prevalence rates of alcohol consumption, alcohol abuse, and drinking and driving behaviors compared to women across all macroregions of Brazil and during all periods analyzed. Differences in alcohol consumption between men and women are well-documented and can be attributed to sociocultural and biological factors^33–35^. Social beliefs regarding gender norms may play a role in shaping these differences in consumption^8^. Conversely, women appear to be at a greater risk of alcohol abuse following traumatic events compared to men^36^. Given this, monitoring women's alcohol consumption patterns in the years following the COVID-19 pandemic is essential.

The study has several limitations that should be considered when interpreting the results. Pre-pandemic data were collected retrospectively during the 2022 Covitel survey, with the first quarter of 2020 designated as the pre-pandemic period. Consequently, the variable "alcohol consumption" may be subject to recall bias. Additionally, a validated instrument was not employed to measure the prevalence and patterns of alcohol consumption. The prevalence of alcohol consumption was based on self-reported data regarding the habit of consuming alcoholic beverages at least once in recent months. However, frequency and quantity of consumption are more accurately captured by the variable measuring abusive alcohol consumption. Despite these limitations, the findings provide valuable insights into alcohol consumption in Brazil during the first quarters of 2022 and 2023.

Although a lower prevalence of reported alcohol consumption was observed in the first quarter of 2022, no reduction was noted in the prevalence of binge drinking or drinking and driving in the first quarter of 2023. The long-term effects of pandemics on alcohol consumption remain largely unknown; however, evidence suggests that traumatic events often lead to increased alcohol consumption in the years that follow. With the official end of the COVID-19 pandemic in 2023, continued monitoring of alcohol consumption should remain a priority for government agencies to support the achievement of the Sustainable Development Goals.
