# Supplementary material for: Abusive consumption of alcoholic beverages: results from COVITEL, the Telephone Survey of Risk and Protective Factors for Noncommunicable Chronic Diseases, 2022 and 2023
Source: Rev Bras Epidemiol. 2025 Feb 24;28:e250009. doi: 10.1590/1980-549720250009 (PMC11849995; doi:10.1590/1980-549720250009)
Supplement: Translation

# Translation: Consumo abusivo de bebidas alcóolicas: resultados do Covitel, Inquérito Telefônico de Fatores de Risco para Doenças Crônicas não Transmissíveis, 2022 e 2023

**DOI:** 10.1590/1980-549720250009.2

**Authors:** Roberta de Oliveira Santos, Fernando César Wehrmeister, Pedro Hallal, Eduardo Ribes Kohn, Luciana Monteiro Vasconcelos Sardinha

**Affiliations:** I Vital Strategies, Departamento de Doenças Crônicas Não Transmissíveis – São Paulo (SP), Brasil.; II Universidade Federal de Pelotas, Centro de Equidade – Pelotas (RS), Brasil.; III University of Illinois, Department of Kinesiology and Community Health – Urbana-Champaign, Estados Unidos da América.; IV Universidade Federal de Pelotas, Programa de Pós-Graduação em Educação Física – Pelotas (RS), Brasil.

**Keywords:** Consumo de bebidas alcoólicas, Brasil, Inquéritos epidemiológicos, Estudos transversais

## Abstract

**Objetivo:**

Estimar a prevalência de consumo abusivo de bebidas alcoólicas, hábito de beber e dirigir e relato de consumo de bebidas alcoólicas, comparando os primeiros trimestres de 2022 e 2023.

**Métodos:**

Estudo transversal, de base populacional, com dados secundários obtidos do Inquérito Telefônico de Fatores de Risco para Doenças Crônicas não Transmissíveis (2022 e 2023). A amostra do estudo integrou 9 mil pessoas em cada ano coletado a partir de métodos de discagem aleatória de dígitos e Discagem Direta à Distância (DDD) em linhas de telefonia móvel e fixa. Variáveis autorreferidas para consumo abusivo de álcool, hábito de beber e dirigir e consumo de bebidas alcoólicas foram analisados.

**Resultados:**

Não houve alteração significativa na prevalência do consumo abusivo de álcool ao se comparar os primeiros trimestres de 2022 e 2023. Contudo, diferenças foram observadas no comportamento de beber e dirigir, com redução da prevalência entre indivíduos de 18 a 24 anos de idade de 9,6% (IC95% 4,4–19,8) para 2,2% (IC95% 1,4–3,6) e aumento do comportamento entre aqueles com 12 ou mais anos de estudo — de 6,9% (IC95% 5,5–8,7) para 11,9% (IC95% 10,3–13,6). Os indivíduos do sexo masculino apresentaram maiores prevalências de consumo de bebidas alcoólicas, consumo abusivo de álcool e hábito de beber e dirigir em todas as desagregações analisadas.

**Conclusão:**

a proposição de políticas públicas que inibam o acesso e o consumo de bebidas alcoólicas e os Objetivos de Desenvolvimento Sustentável devem ser tratados com prioridade no Brasil.

## INTRODUÇÃO

O consumo de bebidas alcoólicas trata-se da ingestão de bebidas que contêm etanol, considerado um dos principais fatores de risco para ocorrência de doenças crônicas não transmissíveis, violência e acidentes, causando diretamente ou indiretamente mais de três milhões de mortes ao ano no mundo^1^. Além da morbimortalidade, ocorrem impactos econômicos em saúde e assistência social ligados a aposentadorias precoces, absenteísmo no trabalho e perda de produtividade^1–4^. Estima-se que o consumo total de álcool *per capita* da população mundial com mais de 15 anos tenha passado de 5,5 litros de álcool puro em 2005 para 6,4 litros em 2016^1^. Cerca de 2,3 bilhões de pessoas consumiam álcool em 2018^1^.

Os dados da Pesquisa Nacional de Saúde de 2013 e 2019 indicam aumento no consumo de álcool pela população brasileira^5^. O mesmo inquérito mostra que os homens consomem mais álcool que as mulheres, entretanto, o aumento no período de 2013 para 2019 foi maior entre as mulheres^5^. Além disso, para consumo abusivo de álcool, houve aumento entre 2013 e 2019^6^. Resultados do estudo CONVID comportamentos, no Brasil, apontaram para o aumento do consumo abusivo de álcool durante a primeira onda da COVID-19 em 2020^7–9^. Dados do relatório da Fiocruz sobre bebidas alcoólicas no Brasil indicam consumo de 13 milhões de litros de cerveja e 2,6 milhões de bebidas destiladas em 2021^10^.

Estudos anteriores sobre o consumo de bebidas alcoólicas no Brasil apresentam resultados importantes para o monitoramento desse padrão de comportamento. Nesse sentido, o presente estudo tem como objetivo ampliar a base de conhecimento sobre consumo de bebidas alcoólicas pela população brasileira comparando o consumo abusivo de bebidas alcoólicas e o comportamento de beber e dirigir entre os primeiros trimestres de 2022 e 2023, de acordo com características socioeconômicas e demográficas.

## MÉTODOS

Trata-se de um estudo transversal, de base populacional, com dados secundários obtidos do Inquérito Telefônico de Fatores de Risco para Doenças Crônicas não Transmissíveis (Covitel) 2022 e 2023. O Covitel é um inquérito nacional conduzido pela Vital Strategies Brasil e pela Universidade Federal de Pelotas (UFPel), com o apoio da Umane, do Instituto Ibirapitanga e da Associação Brasileira de Saúde Coletiva (Abrasco). O projeto Covitel foi aprovado pelo Comitê de Ética em Pesquisa da Escola Superior de Educação Física da Universidade Federal de Pelotas (Parecer número 5.125.635, de 25 de novembro de 2021).

A amostra do Covitel foi segmentada em diferentes estratos, levando em consideração a região geográfica (Nordeste, Norte, Sudeste, Sul e Centro-Oeste), o sexo (masculino e feminino), as faixas etárias (18-34; 35-49 e 50 anos ou mais) e o nível de escolaridade (de 0 a 11 anos e 12 anos completos ou mais). Para calcular o peso amostral, de modo a representar a população brasileira e suas regiões, foram utilizados dados do Sistema IBGE de Recuperação Automática (Sidra; tabela 3450, com base na amostra do Censo Demográfico de 2010). Com isso, a população foi estimada em 60 estratos, considerando a combinação de região geográfica (5), sexo (2), faixa etária (3) e escolaridade.

A amostragem foi feita em duas etapas. A primeira consistiu na criação de um cadastro telefônico de linhas residenciais e de celular, realizado por método de discagem aleatória de dígitos, proporcional ao número de linhas por código de Discagem Direta à Distância (DDD) em cada região do país. A segunda etapa consistiu em um sorteio dos indivíduos, sendo que, para cada linha fixa elegível, este foi feito a partir da relação de todos os moradores do domicílio com pelo menos 18 anos de idade, ordenados de forma crescente por idade. No caso das linhas de celular, foi entrevistado o responsável pela linha, caso tivesse idade maior ou igual a 18 anos. O consentimento livre e esclarecido foi obtido oralmente no momento da aplicação da entrevista.

A amostra foi composta por 1.800 indivíduos por macrorregião, totalizando 9 mil indivíduos para cada ano estudado, dos quais metade foi alocada para telefones fixos e a outra metade para celulares. Esse número permite estimar a frequência de qualquer fator de risco na população estudada com coeficiente de confiança de 95% e erro máximo de cerca de três pontos percentuais. Erros máximos de cerca de quatro pontos percentuais são esperados para as estimativas específicas segundo gênero, assumindo-se proporções semelhantes de homens e mulheres na amostra final. Como o Covitel seleciona participantes com base no código DDD associado à linha telefônica, o que é equivalente a uma amostragem por conglomerados, esse fator foi considerado na análise, juntamente com os pesos amostrais. Assim, as estimativas derivadas foram ajustadas tanto para o efeito de conglomerado quanto de ponderação descrita, de modo a gerar valores representativos das populações regionais e nacional. Detalhes sobre a metodologia do estudo foram previamente descritos por Hallal et al.^11^.

As análises de prevalência do consumo abusivo de bebidas alcoólicas e hábito de beber e dirigir entre os primeiros trimestres de 2022 e 2023, foram baseadas nas seguintes variáveis:

- Consumo abusivo de álcool — sendo aqueles que relataram ter consumido 4 doses (mulheres) ou 5 doses (homens) em uma única ocasião, nos 30 dias que antecederam a entrevista;
- Beber e dirigir — sendo aqueles que relataram que, independentemente da quantidade, costumavam dirigir depois de consumir bebida alcoólica.

Além dos desfechos principais, foi analisada a prevalência do relato de consumir bebidas alcoólicas alguma vez no último mês a partir da variável retrospectiva "*Antes do início da pandemia, o(a) Sr(a) costumava consumir bebida alcoólica?*"; e as variáveis "*Atualmente, o(a) Sr(a) costuma consumir bebida alcoólica?*" do inquérito de 2022 e de 2023 do Covitel.

Para a análise sociodemográfica e de estilo de vida, foram consideradas as seguintes características:

- Sexo (masculino e feminino);
- Faixa etária (18-24; 25-34; 35-44; 45-54; 55-64 e 65 ou mais);
- Regiões do Brasil (Norte, Nordeste, Centro-oeste, Sudeste, Sul);
- Escolaridade (0-8; 9-11 e 12 ou mais);
- Cor da pele (branca; preta e parda e outras).

Medidas de frequência relativa (prevalências) e seus respectivos intervalos de confiança de 95% foram calculados. Foram imputados os pesos amostrais utilizando o módulo *survey* para análise de dados complexos baseado nos dados do censo brasileiro de 2010 realizado pelo Instituto Brasileiro de Geografia e Estatística (IBGE). A amostra foi estratificada com base na região geográfica (Nordeste, Norte, Sudeste, Sul e Centro-Oeste), sexo (masculino e feminino), idade (18-34; 35-49 e 50 anos ou mais) e escolaridade (0-11 anos e 12 anos ou mais de escolaridade). Foram feitas adaptações para idade (a faixa etária de 18-19 anos corresponde a 2/5 da categoria de 15-19 anos do IBGE) e escolaridade (foi criada a categoria de 0-11 anos de estudo para quem estava abaixo do ensino médio nas categorias do IBGE). Mudanças significativas na prevalência dos indicadores foram baseadas na não sobreposição do intervalo de confiança. A análise de dados foi realizada no programa estatístico Stata versão 16 (Stata Corp., College Station, TX, USA), e as figuras foram geradas no *software* Equiplot Creator Tool (International Center for Equity in Health — UFPel).

## RESULTADOS

O total de pessoas entrevistadas em cada fase do estudo Covitel (2022 e 2023) foi de 9 mil, distribuídas igualmente entre cada uma das cinco regiões do país. Não houve diferença significativa para consumo abusivo de álcool no primeiro trimestre de 2022 e de 2023. A prevalência total do relato de beber e dirigir foi maior no primeiro trimestre de 2023 (2,6% IC95% 1,9–3,3), comparada ao primeiro trimestre de 2022 (4,9% IC95% 3,7–6,2). Os subgrupos escolaridade ≥12 anos de estudo apresentaram uma prevalência mais alta no período de 2023 (11,9% IC95% 10,3–13,6), comparado ao período de 2022 (6,9% IC95% 5,5–8,7), e a faixa etária de 18 a 24 anos de idade reduziu a prevalência de 9,6% (IC95% 4,4–19,8) no primeiro trimestre de 2022 para 2,2% (IC95% 1,4–3,6) no primeiro trimestre de 2023 (Tabela 1).

**Tabela 1:** Prevalência do consumo abusivo de álcool e de beber e dirigir, comparando o primeiro trimestre de 2022 e de 2023, segundo variáveis demográficas. Covitel, 2022 e 2023.

Características sociodemográficas e de estilo de vida		Consumo Abusivo de Álcool				Beber e Dirigir			
		2022		2023		2022		2023	
		%	IC_95%_	%	IC_95%_	%	IC_95%_	%	IC_95%_
**Sexo**									
	Masculino	26,6	24,4–28,9	28,9	25,8–32,3	7,6	5,8–10,0	10,6	8,2–13,6
	Feminino	15,0	12,7–17,7	15,7	13,7–18,9	3,4	2,3–4,9	2,1	1,5–3,0
**Faixa Etária**									
	18 a 24 anos	25,8	21,2–30,9	32,6	26,4–39,5	9,6	4,4–19,8	2,2 ↓	1,4–3,6
	25 a 34 anos	26,9	23,2–31,0	27,0	22,5–32,0	6,0	3,8–9,3	8,7	6,0–12,3
	35 a 44 anos	23,2	20,5–26,1	23,7	20,1–27,7	7,7	5,9–9,8	9,7	7,0–13,3
	45 a 54 anos	20,1	17,2–23,4	23,6	19,5–28,3	5,1	3,5–7,4	6,7	4,5–10,0
	55 a 64 anos	12,9	10,0–16,5	14,4	11,9–17,3	4,5	2,2–8,8	3,7	2,3–5,8
	65 anos ou mais	5,5	4,3–7,1	5,1	4,0–6,4	2,2	0,8–5,4	1,5	0,9–2,4
**Regiões do país**									
	Norte	22,8	21,5–24,3	19,0	15,3–23,2	6,7	5,0–8,8	6,9	4,6–10,2
	Nordeste	20,0	16,8–23,7	21,2	17,7–25,1	7,5	4,4–12,6	5,4	3,0–9,6
	Centro-Oeste	18,9	15,1–23,2	21,9	17,4–27,2	6,0	3,8–9,3	4,1	2,2–7,4
	Sudeste	21,0	18,1–24,1	23,7	20,8–26,8	5,8	4,0–8,3	6,8	4,9–9,3
	Sul	20,3	17,0–24,1	20,6	18,3–23,1	5,1	2,9–8,8	6,8	4,5–10,3
**Escolaridade**									
	0 a 8 anos	17,1	14,8–19,6	19,8	16,2–24,0	4,6	2,6–8,0	4,9	3,1–7,7
	9 a 11 anos	22,5	19,8–25,5	22,9	19,5–26,7	7,6	5,0–11,4	4,6	2,9–7,2
	12 ou mais	26,6	24,3–29,1	26,6	25,0–28,2	6,9	5,5–8,7	11,9 ↑	10,3–13,6
**Cor da Pele**									
	Branca	18,8	16,5–21,4	19,8	16,6–23,3	7,0	5,5–8,8	7,8	5,9–10,4
	Preta e Parda	22,5	20,2–24,9	18,8	16,0–21,9	5,7	4,0–8,0	4,1	2,8–5,8
	Outras	16,1	10,6–23,7	16,9	11,4–24,2	4,7	1,3–15,8	4,9	2,3–10,1
**Total**		20,6	18,9–22,4	22,1	20,2–24,0	2,6	1,9–3,3	4,9 ↑	3,7–6,2

↓ diminuição da prevalência no período do primeiro trimestre de 2023;

↑ aumento da prevalência no período do primeiro trimestre de 2023.

Indicadores sem símbolo ao lado indicam que não houve alteração na prevalência dos indicadores de beber e dirigir e consumo abusivo de álcool baseado na sobreposição dos intervalos de confiança.

Entre os grupos estratificados, a prevalência do consumo abusivo de bebidas alcoólicas foi significativamente maior entre os homens no período de 2022 (26,6% IC95% 24,4–28,9) e no período de 2023 (28,9% IC95% 25,8–32,3), comparados às mulheres no período de 2022 (15% IC95% 12,7–17,7) e no período de 2023 (15,7% IC95% 13,7–18,9). Pessoas com 65 anos ou mais tiveram prevalências significativamente mais baixas de consumo abusivo de bebidas alcoólicas comparadas aos subgrupos de idade <65 anos no primeiro trimestre de 2022 e 2023, enquanto as pessoas entre 0 e 8 anos de estudo tiveram prevalências significativamente menor somente no primeiro trimestre de 2022, comparadas às pessoas com nível de escolaridade maior (Tabela 1).

Para o relato de beber e dirigir, a prevalência foi significativamente maior entre os homens no período de 2022 (7,6% IC95% 5,8–10,0) e no período de 2023 (10,6% IC95% 8,2–13,6), comparados às mulheres no período de 2022 (3,4% IC95% 2,3–4,9) e no período de 2023 (2,1% IC95% 1,5–3,0). Os subgrupos mais extremos de idade tiveram prevalências menores de beber e dirigir, comparados aos demais subgrupos somente no período de 2023. Pessoas com ≥12 anos de estudo no período de 2023 apresentaram prevalência de beber e dirigir significativamente maior (11,9% IC95% 10,3–13,6), comparadas às pessoas com 9 a 11 anos de estudo (4,6% IC95% 2,9–5,8) e às pessoas com 0 a 8 anos de estudo (4,9% IC95% 2,9–7,2), conforme consta na Tabela 1.

O relato de consumo de bebidas alcoólicas no Brasil, analisado de forma retrospectiva em janeiro de 2020, apresentou prevalência maior (44,2%; IC95% 41,8–46,6) quando comparado ao período de 2022 (37,8% IC95% 35,7–40,0). Entretanto, a prevalência desse indicador no período de 2023 não apresentou diferença significativa comparada aos outros dois momentos (41,5% IC95% 39,2–43,9) (Figuras 1 a 4).

**Figura 1:** Prevalência do consumo de bebida alcoólica, segundo Brasil e regiões do país, no 1° trimestre de 2020, no 1° trimestre de 2022 e no 1° trimestre de 2023. Covitel, 2022 e 2023.

**Figura 2:** Prevalência do consumo de bebida alcoólica, segundo sexo, no 1° trimestre de 2020, no 1° trimestre de 2022 e no 1° trimestre de 2023. Covitel, 2022 e 2023.

**Figura 3:** Prevalência do consumo de bebida alcoólica, segundo faixa etária, no 1° trimestre de 2020, no 1° trimestre de 2022 e no 1° trimestre de 2023. Covitel, 2022 e 2023.

**Figura 4:** Prevalência do consumo de bebida alcoólica, segundo escolaridade, no 1° trimestre de 2020, no 1° trimestre de 2022 e no 1° trimestre de 2023. Covitel, 2022 e 2023.

## DISCUSSÃO

O presente estudo analisou a prevalência de consumo abusivo de bebidas comparando os primeiros trimestres de 2022 e 2023 no Brasil. Não foi observada mudança significativa na prevalência de consumo abusivo de álcool nos períodos do estudo Covitel. A literatura mostra que indivíduos com esse perfil de consumo são normalmente menos motivados a mudarem seus comportamentos devido ao ajuste do organismo aos resultados negativos associados às intoxicações repetidas^12,13^. Nos Estados Unidos, uma coorte com mais de 2 milhões de pessoas observou que o consumo abusivo de álcool diminuiu de 15,5% para 14,6% durante o primeiro ano da pandemia, aumentou para 15,2% no segundo ano e depois diminuiu para 14,9% no período de março de 2022 a fevereiro de 2023^14^, mantendo certa estabilidade.

Entretanto, as prevalências semelhantes observadas nos dados de consumo abusivo de bebidas alcoólicas do Covitel não devem ser tratadas como algo positivo, mesmo se tratando de um período que engloba, em parte, a pandemia de COVID-19. O período observado é relativamente curto e as consequências da pandemia podem causar aumento nesse indicador. Evidências indicam que, nos anos subsequentes aos eventos traumáticos e às recessões econômicas, o consumo de bebidas alcoólicas tende a aumentar em decorrência dos episódios de estresse e ansiedade^15–18^.

O consumo de bebidas alcoólicas é tratado como uma prioridade de saúde em nível mundial, sendo abordado entre os Objetivos de Desenvolvimento Saudável (ODS) com a meta de "fortalecer a prevenção e o tratamento do abuso de substâncias, incluindo abuso de drogas e uso prejudicial de álcool"^19^. Anteriormente à pandemia, a Organização Mundial de Saúde (OMS) organizou um pacote técnico com cinco estratégias de impacto que podem ajudar os governos a reduzirem o uso prejudicial de álcool e suas consequências sociais, econômicas e de saúde^20^. O Brasil deve abordar o consumo de bebidas alcoólicas com a importância que o tema merece para a saúde, considerando as recomendações globais. Nesse sentido, o Plano Nacional de Políticas sobre Drogas (2022-2027) poderá auxiliar no enfrentamento ao uso abusivo de bebidas alcoólicas no Brasil^21^.

Quanto à prevalência do hábito de beber e dirigir no primeiro trimestre de 2023, indivíduos de 18 a 24 anos reduziram tal indicador, enquanto pessoas com 12 anos ou mais de estudo aumentaram a prevalência, comparado ao período de 2022. Não há evidências claras que justifiquem essas mudanças nesses subgrupos. Vingilis e colaboradores sugeriram que a pandemia de COVID-19 tinha potencial de impactar de forma diferente determinados grupos sociais^22^. Uma hipótese para a diminuição do hábito de beber e dirigir entre os mais jovens é a perda de emprego e renda, impondo a esse subgrupo piores condições econômicas. As pessoas com 12 ou mais anos de escolaridade podem ter sofrido menos impacto econômico em decorrência da pandemia. Dados da Pesquisa Nacional de Saúde (PNS) mostram associação entre beber e dirigir e pessoas do sexo masculino com maior renda^23^.

Estudos prévios identificaram que um dos preditores do hábito de beber e dirigir é a experiência de não receber punições^24,25^, ou seja, envolver-se em comportamentos de condução sob o efeito do álcool sem ser capturado e punido posteriormente^26^. Os condutores sem histórico do hábito de beber e dirigir, que iniciaram esse comportamento, provavelmente evitaram punições, o que pode promover o envolvimento contínuo desse comportamento de risco no futuro^27^. Entretanto, reduzir pela metade as mortes e os ferimentos globais por acidentes em estradas é um componente dos Objetivos de Desenvolvimento Sustentável^19^ que pode ser cumprido a partir de medidas de conscientização sobre o hábito de beber e dirigir.

A prevalência de relato do costume de consumir bebidas alcoólicas no Brasil foi menor no primeiro trimestre de 2022, comparada ao momento antes da pandemia no primeiro trimestre de 2020. Em contrapartida, o estudo ConVid no Brasil observou que o hábito de consumir bebidas alcoólicas durante a pandemia aumentou, comparado ao período pré-pandemia^8^. Entre adolescentes, o estudo ConVid observou que a prevalência do costume de consumir bebidas alcoólicas reduziu significativamente do período pré-pandemia para momentos durante a pandemia^28^. Bebidas alcoólicas foram consumidas em média um dia a mais por mês por 3 em cada 4 adultos durante o primeiro ano de pandemia nos Estados Unidos^29^. No México e na Colômbia, a prevalência do consumo de bebidas alcoólicas diminuiu durante a pandemia, apresentando redução mensal na pontuação do *Alcohol Use Disorders Identification Test* (AUDIT), de 1,9% no México e 1,5% na Colômbia^30^.

Durante crises sanitárias, são possíveis dois cenários opostos em relação ao consumo de álcool: no primeiro, devido ao aumento de estresse em decorrência do isolamento social, pode ser observado aumento do consumo de bebidas alcoólicas; em um segundo cenário, as medidas de distanciamento geram dificuldades de acesso aos locais de venda, a perda de emprego e renda gera redução da capacidade financeira para a compra, e as medidas de restrição proíbem reuniões sociais, restringindo o consumo^31,32^. Os estudos do Covitel foram desenvolvidos em períodos da pandemia de COVID-19, nesse sentido, mudanças no padrão de consumo durante esse período poderiam ser observadas.

Indivíduos do sexo masculino apresentaram prevalências de consumo de bebidas alcoólicas, consumo abusivo de álcool e comportamento de beber e dirigir mais elevados, quando comparados às mulheres em todas as macrorregiões do Brasil e em todos os períodos analisados. Diferenças no consumo de bebidas alcoólicas entre homens e mulheres são esperadas, considerando fatores socioculturais e biológicos^33–35^. A relação entre consumir álcool e crenças sociais sobre normas de gênero pode influenciar essa diferença de consumo^8^. Por outro lado, mulheres parecem ter maior risco de uso abusivo de álcool após eventos traumáticos, quando comparadas aos homens^36^. Nesse sentido, nos anos subsequentes à pandemia de COVID-19, se faz necessário acompanhar o comportamento das mulheres em relação aos padrões de consumo de bebidas alcoólicas.

O estudo possui algumas limitações que devem ser consideradas no momento da interpretação dos resultados. Os dados pré-pandemia foram obtidos de forma retrospectiva durante o inquérito de 2022 do Covitel, considerando como período pré-pandemia o primeiro trimestre de 2020. Com isso, a variável "consumo de bebidas alcoólicas" pode sofrer de viés de recordatório. Não foi utilizado um instrumento validado para aferir a prevalência e os padrões de consumo de álcool. A prevalência de consumo de álcool foi baseada no relato do costume de consumir bebidas alcoólicas pelo menos alguma vez nos últimos meses. Entretanto, a frequência e a quantidade de consumo são mais bem explicadas pela variável sobre consumo abusivo de bebidas alcoólicas. Apesar dessas limitações, esses dados contribuem para nosso conhecimento sobre o consumo de álcool no primeiro trimestre de 2022 e 2023 no Brasil.

Apesar de observada menor prevalência do relato de consumo de bebidas alcoólicas no primeiro trimestre de 2022, não foi observada diminuição da prevalência de consumo abusivo de álcool no primeiro trimestre de 2023, nem no hábito de beber e dirigir. Pouco se sabe sobre os efeitos de longo prazo das pandemias no consumo de álcool; entretanto, eventos traumáticos tendem a aumentar o consumo de bebidas alcoólicas nos anos subsequentes. Com o fim da pandemia de COVID-19 em 2023, manter a vigilância sobre o consumo de álcool deve ser um compromisso contínuo dos órgãos governamentais para o cumprimento dos Objetivos de Desenvolvimento Sustentável.
